# Identification of barley powdery mildew resistances in gene bank accessions and the use of gene diversity for verifying seed purity and authenticity

**DOI:** 10.1371/journal.pone.0208719

**Published:** 2018-12-07

**Authors:** Antonín Dreiseitl, Marta Zavřelová

**Affiliations:** 1 Department of Integrated Plant Protection, Agrotest Fyto Ltd., Kroměříž, Czech Republic; 2 Department of Plant Genetics and Breeding, Agrotest Fyto Ltd., Kroměříž, Czech Republic; Julius Kuhn-Institut, GERMANY

## Abstract

Human activities including those in crop gene banks are subject to errors, especially during seed multiplication and maintenance of seed germination. Therefore, the most serious problem of gene banks is authenticity of the accessions and their genotypic purity. There are many methods for determining the identity of varieties, but comparisons between current data and past records are not easy since the latter are often missing. Breeding barley resistant to powdery mildew caused by *Blumeria graminis* f. sp. *hordei* (*Bgh*) was traditionally based on incorporating major genes into new varieties and the results have been published. Our goal was to identify resistance genes to powdery mildew in accessions of the Czech spring barley core collection and compare these data with earlier information to establish the authenticity of the accessions. Two hundred and twenty-three accessions of the collection including 665 single plant progenies were tested. Sixty-four selected reference isolates of *Bgh* representing the world diversity of the pathogen were used for resistance tests. Twenty-two known resistance genes were postulated either separately or in combinations. In the collection, 151 homogeneous accessions were found, but the resistances of nine of them were inconsistent with published data and in 12 accessions their authenticity is doubtful. The remaining 72 accessions were heterogeneous and comprised 176 resistance genotypes, 54 of which were probably mechanical admixtures of other varieties. There are several pathogens of cereals, e.g. rusts and mildews, against which many resistance genes in host crops have also been exploited. Knowledge of these resistances can assist in maintaining pure and genuine stocks in gene banks. Seed purity and the authenticity of accessions can subsequently be checked with more advanced methods.

## Introduction

Barley (*Hordeum vulgare* L.) is one of most important cereal crops in the world. Genetic resistance in cultivated plant species plays an essential part in disease management and plant genetic resources are key to improving crops. Gene banks contain vast collections of varieties, but there are often groups of similar genotypes. Therefore, model collections, so-called core collections, have been created [1–3], which should provide as much genetic diversity as possible in a limited number of genotypes.

Human activity can result in errors and in gene banks these can cause problems relating to seed multiplication when each reproductive cycle comprises several operations where genotype contamination can occur especially after repeated cycles. To counter such errors gene banks implement standardized procedures, but in the past such procedures were not sufficiently elaborated and even with these techniques errors are still possible.

Varieties deposited in gene banks are mostly used for research and breeding and any genotype contamination creates more work for investigators; unintentional use of admixtures or misnamed varieties compromises the results [4,5]. Hence, the authenticity and genotypic purity of accessions in gene banks is essential. There are many methods available for determining varietal identity [6–8], including sequencing methods [9,10]. However, such refined methods may create more confusion if they are used on unverified varieties.

Powdery mildew, caused by the fungus *Blumeria graminis* (D.C.) Golovin ex Speer f. sp. *hordei* Em. Marchal (*Bgh*), is a worldwide disease that can cause frequent epidemics of barley particularly in Central Europe [11]. To combat this, genetic resistance is an efficient and environmentally acceptable way of limiting its effect on yield and quality.

Breeding barley resistant to mildew, particularly in Europe, was traditionally based on major genes. The sources of resistance were at first landraces [12–14], but were later superseded by wild barley (*Hordeum vulgare* subsp. *spontaneum*) obtained from its centre of diversity [15]. The utilization of resistance genes in breeding has been closely monitored [16–18], summarized [19] and subsequently updated [20,21]. With the change in gene bank personnel and management it is now opportune to reconsider the current state of stored accessions.

Our goal was, therefore, i) to check the homogeneity of accessions included in the Czech core collection of spring barley regarding major resistance genes to powdery mildew, ii) to identify resistance genes to powdery mildew in the accessions, and iii) based on previously published resistance data, to verify the authenticity of the accessions and, in the case of any inconsistencies, to identify those accessions of doubtful authenticity.

## Materials and methods

### Plant material and pathogen isolates

We tested all 223 accessions of the Czech spring barley core collection including 665 single plant progenies. For resistance tests we used 64 selected reference isolates of *Bgh* from our gene bank of the pathogen collected in 12 countries in all non-polar continents over a period of 63 years (1953–2016) which represent the world diversity of the pathogen (S1 Table). Before inoculation we checked isolates for their purity, verified the correct pathogenicity phenotype on standard barley lines [22] and multiplied on leaf segments of susceptible variety Stirling [23].

### Testing procedure

We sowed about 60 seeds of each accession in two pots (80 mm diameter) filled with a garden peat substrate and placed them in a mildew-proof greenhouse under natural daylight. Then we cut leaf segments 15 mm long from the central part of healthy fully-expanded primary leaves when second leaves were emerging. We placed three segments adjacent to each other along with four segments of the susceptible Stirling oriented diagonally and with adaxial surfaces facing upward in a 150 mm Petri dish on water agar (0.8%) containing benzimidazole (40 mg^-L^)—a leaf senescence inhibitor. For testing single plant progenies, we planted seed from one spike in a pot and used a leaf segment from each.

For isolate inoculation, we used a cylindrical metal settling tower of 150 mm diameter and 415 mm in height and we placed a dish with leaf segments at the bottom of the tower. We shook conidia of each isolate from a leaf segment of the susceptible variety with fully developed pathogen colonies onto a square piece (40 x 40 mm) of black paper to visually estimate the amount of inoculum deposited. Then we rolled the paper to form a blowpipe and we blew conidia of an isolate through a side hole of 13 mm diameter in the upper part of the settling tower over the Petri dish at a concentration of ca. 8 conidia mm^-2^. The dishes with inoculated leaf segments were incubated at 18±2°C under artificial light (cool-white fluorescent lamps providing 12 h light at 30±5 μmol m^-2^ s^-1^).

### Evaluation

Eight days after inoculation, we scored response types (RT = phenotype of barley variety x pathogen isolate interaction) on the central part of the adaxial side of leaf segments on a scale 0–4, where 0 = no visible mycelium or sporulation, and 4 = strong mycelial growth and sporulation on the leaf segment [17]. An RT0(3) representing RT0 with presence of a few mildew colonies was added [24]; generally, RTs 0–3 and 0(3) were considered resistant, but a typical RT of each resistance gene was also taken into account. We tested each accession with a minimum of two replications. If there were significant differences in RTs between replications, we carried out additional tests. A set of 64 RTs provided a response type array (RTA) for each accession. Based on the gene-for-gene model [25], we postulated the resistance genes in accessions by comparing the RTAs with previously determined RTAs of standard barley genotypes possessing known resistance genes.

### Assessment of results

The authenticity of genotypes was assessed by comparing the results of their resistance recorded in this project with published data obtained around the time of registration of commercial varieties. The basic source of information was a catalogue containing information on the registration of these mostly European varieties [19]. In addition, information relating to their pedigree and the year of their registration or the acquisition date by the gene bank was used.

## Results

First tests of 223 spring barley accessions showed that 90 of them had pure seed and were homogeneous. For each of the remaining 133 heterogeneous accessions we harvested five single plants and 665 progenies were re-tested. In 61 varieties, all five progenies had identical RTAs, although in the original accessions they were heterogeneous. In the remaining 72 sets different RTAs were found, which represented 176 genotypes. In 45 sets we detected two different genotypes, in 22 sets three and in 5 sets four genotypes. There were 327 accession × powdery mildew resistance genotypes in the core collection (Table 1).

**Table 1:** Specific resistance genes against *Blumeria graminis* f. sp. *hordei* in 223 accessions of varieties included in the Czech core collection of spring barley.

Code^a^	Variety	Country^b^	*Ml* resistance gene	Category^c^
0446	Abyssinian 1102	ETH	*a8*, *He2*	e
0446	Abyssinian 1102	ETH	*a7*, *g*	e
0446	Abyssinian 1102	ETH	none	e
0448	Abyssinian 1113	ETH	*a6*	c
2043	Abyssinian 21	ETH	*a8*, *k1*	c
2043	Abyssinian 21	ETH	*g*, *k1*	c
1231	Adonia	DEU	*a6*, *h*, *ra*	a
1231	Adonia	DEU	*p1*	a
1231	Adonia	DEU	*p1*, *at*	a
1231	Adonia	DEU	*a12*, *u*	e
0760	Agio	NLD	none	b
2182	Akcent	CSK	*a7*, *La*	a
2182	Akcent	CSK	*a3*	e
1986	Akta Abed	DNK	*a7*	a
0911	Algerian	DZA	*a1*, *at*	a
2202	Amalia	AUT	*a9*, *g*, *u*	a
2342	Amulet	CSK	*a13*, *La*	a
1437	Apex	NLD	*mlo*	a
1103	Aramir	NLD	*a12*, *g*	a
0824	Archer	GBR	*a8*	e
2240	Arra	FIN	*a8*	b
0738	Asplund	SWE	*a8*	b
0334	Asse	DEU	*ra*, *u*	e
0334	Asse	DEU	*a8*	e
1537	Athos	FRA	*a12*, *g*	a
2343	Atribut	CSK	*mlo*	a
2245	Attiki	GRC	*p1*	a
2245	Attiki	GRC	*p1*, *g*	a
2245	Attiki	GRC	*g*, *La*	e
0754	Aurore	FRA	*a8*	b
0969	Australische Fruehe	AUS	*a8*	b
0969	Australische Fruehe	AUS	*Ch*	b
1481	Azuma Mugi	JPN	*Ru2*	b
1953	Bai Liu Leng	CHN	*u*	b
0939	Balder Ohra	SWE	*Ch*	a
2140	Ballerina	DEU	*a12*, *g*, *k1*	a
0516	Bavaria Ackermanns	DEU	*Ch*, *He2*	c
2162	Beladi	EGY	*a8*, *u1*	d
2162	Beladi	EGY	*a8*, *u2*	d
1171	Beta 6 Kora	HUN	*a8*	b
1171	Beta 6 Kora	HUN	*k1*	c
0667	Bethge II	DEU	*a8*	b
0557	Bethges III	DEU	*a8*	b
1024	Bigo	NLD	*Ch*	b
0719	Binder Abed	DNK	*g*, *He2*	a
2012	Bingo Carlsberg	DNK	*a13*	a
2012	Bingo Carlsberg	DNK	*a12*, *g*	e
2012	Bingo Carlsberg	DNK	*a12*, *g*, *La*	e
2040	Black Hull-Less	USA	*u*	a
2307	Blondie	SWE	*a12*, *La*	a
2307	Blondie	SWE	*a12*, *u*	a
2307	Blondie	SWE	*a7*, *La*	e
1083	Bode	NOR	*a8*	b
0012	Bohatyr	CSK	*a8*	b
1014	Bolivia	USA	*u*	d
0070	Branisovicky C	CSK	*a8*	b
0070	Branisovicky C	CSK	*Ch*, *He2*	b
2434	Brenda	DEU	*mlo*	a
0576	Breustedts Harzer Imperial	DEU	*a8*	b
2516	Buck	CAN	none	d
0718	Carlsberg	DNK	*a8*	a
2298	Cask	GBR	*a13*	c
0057	Celechovicky Hanacky	CSK	*a8*, *He2*	b
0636	Ceres	FRA	*a8*	b
0636	Ceres	FRA	none	b
0636	Ceres	FRA	*g*	c
0637	Ceresia Ackermanns	DEU	*g*	a
0637	Ceresia Ackermanns	DEU	*a9*, *g*	e
0637	Ceresia Ackermanns	DEU	*Ch*	e
0851	Clermont	FRA	*Ch*	b
0908	Club Marriout	EGY	*a8*, *u*	d
0908	Club Marriout	EGY	*ra*, *Ch*	d
1472	Combi	DEU	*a7*, *g*	a
1472	Combi	DEU	*a9*	e
0757	Commander	FRA	*a8*, *u*	d
2408	Cooper	GBR	*a1*, *La*	a
2452	Cork	GBR	*a1*, *Ab*	a
0241	Danubia Ackermanns	DEU	none	a
0241	Danubia Ackermanns	DEU	*Ch*	b
0241	Danubia Ackermanns	DEU	*a8*	e
0241	Danubia Ackermanns	DEU	*g*	e
0347	Denso	DNK	*a8*	a
2051	Deuce	CAN	*a7*, *u*	d
0166	Diamant	CSK	*a8*	b
0166	Diamant	CSK	*a8*, *He2*	b
0166	Diamant	CSK	*a7*	e
0166	Diamant	CSK	*mlo*	e
2098	Dinky	BEL	*a9*, *k1*, *La*	a
2098	Dinky	BEL	*a8*, *k1*	c
0032	Dobrovicky Starocesky	CSK	*a8*	b
0032	Dobrovicky Starocesky	CSK	*a8*, *He2*	b
0032	Dobrovicky Starocesky	CSK	*Ch*	b
0032	Dobrovicky Starocesky	CSK	none	b
0538	Dometzkoer Paradies Nackte	DEU	*a8*, *He2*	b
0512	Donaria Ackermanns	DEU	*Ch*, *He2*	a
0899	Doneckij 9	SUN	*a12*	d
0065	Dregeruv	CSK	*a8*	b
0123	Druzba	SUN	*a7*, *g*, *La*	c
0123	Druzba	SUN	*a7*, *h*, *La*	c
0123	Druzba	SUN	*g*	c
2146	Duckbill	GBR	none	d
0900	Early Chevalier	CAN	*Ch*	b
0900	Early Chevalier	CAN	none	b
0575	Ebstorfer Nacktgerste	DEU	none	b
0527	Egelfinger Monarchia	DEU	*a8*	b
0075	Ekonom	CSK	*a8*	b
0780	Emir	NLD	*a8*	e
0780	Emir	NLD	none	e
0450	Entresole	BOL	*a8*, *u*	b
0450	Entresole	BOL	none	d
1350	Esperance No. 227/1960	FRA	*a8*	c
2528	Falcon	CAN	*Ch*	d
2528	Falcon	CAN	none	d
1128	Franzista	DEU	*a8*, *La*	d
0759	Frisia Breustedts	DEU	*a8*, *u*	d
0657	Gerda	DEU	*a7*, *g*, *k1*	e
0657	Gerda	DEU	*a8*	e
0657	Gerda	DEU	*g*	e
0765	Glattgrannige von Vilmorin	USA	none	b
0765	Glattgrannige von Vilmorin	USA	*g*	c
1003	Golden Promise	GBR	*a8*	a
1607	Goldmarker	GBR	*a6*, *La*	a
2244	Grammos	GRC	*Ch*	b
0517	Granat Breustedts	DEU	*a8*, *u*	c
0413	Gull Svalofs	SWE	*Ch*	a
0507	Hadostreng	DEU	*a8*, *u*	d
0523	Haisa I Heines	DEU	*Ch*	b
0523	Haisa I Heines	DEU	none	b
0090	Hana	CSK	*a8*, *He2*	a
0090	Hana	CSK	*g*, *He2*	c
0002	Hanacky Jubilejni	CSK	*a8*	b
0002	Hanacky Jubilejni	CSK	*a8*, *He2*	b
0013	Hanacky Kargyn	CSK	*a8*	b
0689	Hanna	CSK	*g*	a
0168	Harbine	USA	none	b
2572	Heris	CZE	*mlo*	a
2024	Hermine	FRA	*a7*, *g*, *k1*	a
1169	Hero	USA	*a8*, *u*	b
1169	Hero	USA	*Ch*	b
1169	Hero	USA	none	b
1255	Hiproly	ETH	none	b
1255	Hiproly	ETH	*a12*	d
1993	Hockey	GBR	*a12*, *La*	a
0854	Hunter	IRL	*a8*	a
0876	Husky	CAN	*Ch*	b
0876	Husky	CAN	none	b
2349	Chariot	GBR	*mlo*	a
2126	Charkovskii 91	SUN	*a7*, *k1*	c
0923	Chevallier	GBR	*a8*	b
0923	Chevallier	GBR	*Ch*	b
0923	Chevallier	GBR	none	b
1152	Chevron	USA	*g*, *h*	a
1152	Chevron	USA	*h*, *u*	a
0023	Chlumecky	CSK	*a8*	b
2188	Icare	FRA	*a13*, *g*, *La*	c
0529	Isaria Ackermanns	DEU	*Ch*, *He2*	b
0671	Isaria Nova	DEU	*a8*, *He2*	b
0671	Isaria Nova	DEU	*a6*	e
0671	Isaria Nova	DEU	*a6*, *g*	e
2038	Ishtar	CHN	*a8*	b
2038	Ishtar	CHN	none	b
2164	Izmir 9	TUR	*g*	c
2164	Izmir 9	TUR	*g*, *at*	c
0158	Jantar	CSK	*g*	a
0158	Jantar	CSK	*a8*	e
2395	Jelen	YUG	*a7*, *g*, *La*	a
0132	Kasticky	CSK	*a8*	b
1478	Kilta	FIN	none	d
2508	Klinta	LVA	*a8*, *La*	a
0085	KM 1192	CSK	*a8*, *La*	e
0085	KM 1192	CSK	*a8*	e
0515	Kneifels Vollkorn	DEU	*a8*, *He2*	b
0089	Koral	CSK	*a13*, *g*	a
0093	Krajova St. Hrozenkov	CSK	*a8*	b
0104	Krystal	CSK	*a13*, *g*	a
0568	Lada	DDR	*a12*	a
0568	Lada	DDR	*a8*, *He2*	e
2026	Lapac	YUG	*Ch*	b
2026	Lapac	YUG	none	b
2026	Lapac	YUG	*a9*, *g*	e
0826	Lion	USA	none	b
2460	Logan	USA	*a8*, *k1*	a
2460	Logan	USA	*a1*, *g*	e
1428	Lud	GBR	*g*, *La*	a
2340	Lumar	CSK	*a1*, *g*, *k1*	a
1507	Lyallpur 3647	IND	*a7*, *k1*	a
0704	Maja Abed	DNK	*a8*	a
0704	Maja Abed	DNK	*g*, *He2*	e
2153	Malebo	AUS	*a8*, *k1*	e
1002	Malteria Heda	ARG	*a8*	a
1002	Malteria Heda	ARG	*a6*, *La*	e
1002	Malteria Heda	ARG	*a7*, *g*, *k1*	e
2034	Manchuria	USA	none	a
0766	Mansholts Tweerijige	NLD	none	b
0745	Maskin	NOR	*a8*, *He2*	e
0865	Maythorpe	GBR	*a8*	a
0592	Mehltauresistente II Firlbecks	DEU	*g*	a
0592	Mehltauresistente II Firlbecks	DEU	*g*, *He2*	a
0147	Merkur	CSK	*g*	a
0699	Midas	GBR	*a6*	a
1155	Monte Cristo	IND	*a9*, *k1*	a
1155	Monte Cristo	IND	*a1*	e
1155	Monte Cristo	IND	*a13*	e
2047	Murasski Mochi	USA	*u*	d
1216	Nadja	DDR	*a7*, *k1*, *La*	c
1216	Nadja	DDR	*a8*	e
2313	Nagrad	POL	*g*, *La*	a
2313	Nagrad	POL	*a13*	e
2456	Namoi	AUS	*Ch*	a
0042	Nolc-Dregeruv Imperial A	CSK	*a8*	b
0086	Nolc-Dregeruv Velerany	CSK	*a8*	b
2220	Nomad	GBR	*a9*, *La*, *u*	a
0004	Novodvorsky Hanacky	CSK	*a8*	b
2394	Novosadski 406	YUG	*a13*, *g*	d
2394	Novosadski 406	YUG	*a7*	d
2394	Novosadski 406	YUG	*g*, *La*	d
0074	Novum	CSK	*a13*, *g*	a
2285	Nugget	GBR	*a13*, *La*	a
1025	Oderbrucker	USA	none	a
0514	Oderlongauner Kneifelgerste	DEU	*a8*	b
0514	Oderlongauner Kneifelgerste	DEU	none	b
2329	Odesskij 131	SUN	*a7*, *g*, *La*	c
0201	Odesskij 9	SUN	*g*, *La*	e
2015	Odissej	SUN	*a12*	c
2015	Odissej	SUN	*a13*, *g*	e
0792	Olli	FIN	none	b
2076	Olont	MNG	*a8*	b
2112	Omskij 13709	SUN	*a7*, *k1*	c
2112	Omskij 13709	SUN	*mlo*	e
0101	Opal	CSK	*a8*	e
0101	Opal	CSK	*a7*, *La*	e
0005	Opavsky Kneifl	CSK	*a8*	b
1273	Otra	FIN	*a7*, *La*	e
0621	Otterbacher	AUT	*a8*, *He2*	b
1027	Palestine 10	EGY	*a8*, *k1*, *La*	a
2365	Pannonia	AUT	*mlo*	a
1467	Patty	FRA	*a12*, *g*	a
2371	Pax	CSK	*a13*, *La*	a
0848	Peatlant	USA	none	b
0935	Peruvian	USA	*at*	a
2292	Phantom	DDR	*a13*, *g*	a
2093	Pirogovskij	SUN	*a8*	c
0680	Plena	DDR	*g*	c
0680	Plena	DDR	*g*, *He2*, *Lo*	c
0680	Plena	DDR	*g*, *Lo*	c
0821	Plumage Archer	GBR	*a8*	e
2135	Princesse	DEU	*a3*, *g*, *La*	a
2135	Princesse	DEU	*a3*, *g*	b
2135	Princesse	DEU	*g*	b
0834	Prior	AUS	*a8*	a
2524	Prosa	AUT	*g*, *u*	a
0079	Proskowtzuv	CSK	*a8*, *He2*	b
0079	Proskowtzuv	CSK	*g*	e
0866	Provost	GBR	none	a
0617	Pumper 6 ZLG	AUT	*h*	b
1243	Quantum	AUT	*g*, *u*	a
1243	Quantum	AUT	*a12*, *La*, *g*	e
0605	Ragusa 415	YUG	*ra*, *Lo*	a
0605	Ragusa 415	YUG	*p1*, *ra*, *Lo*	e
0017	Ratborsky	CSK	*a8*	b
1915	Research	AUS	*a8*	a
2101	Roxane	FRA	*a12*, *g*, *u*	a
1299	RTG Valticky	CSK	*a8*, *He2*	b
1299	RTG Valticky	CSK	*a12*	e
1299	RTG Valticky	CSK	*a13*	e
1299	RTG Valticky	CSK	*g*	e
0059	Rubin	CSK	*a1*	a
1622	Rupee	IND	*u*	e
0756	Sarah	FRA	none	a
2354	Saxo	DNK	*mlo*	a
0594	Saxonia Malz Imperial	DEU	*a8*	b
0163	Selekcni Hanacky VIII.	CSK	*g*, *h*	b
0163	Selekcni Hanacky VIII.	CSK	*g*, *He2*	b
0163	Selekcni Hanacky VIII.	CSK	*at*	e
0054	Semcicky Hospodarsky	CSK	*a8*	b
0054	Semcicky Hospodarsky	CSK	none	b
0054	Semcicky Hospodarsky	CSK	*a1*	e
2266	Senor	DNK	*a13*	a
0626	Schwarzenberg Gerste 21	DEU	*a6*, *g*	c
1285	Sinaji Mugi	JPN	none	b
0197	Sladar	CSK	*a8*	b
0197	Sladar	CSK	none	b
0008	Slovensky Dunajsky Trh	CSK	*a8*	b
0055	Spartan	CSK	*a9*, *k1*	a
0055	Spartan	CSK	*a6*, *g*	e
0702	Stella Svalofs	SWE	*a8*	b
0702	Stella Svalofs	SWE	none	b
1054	Stephan	CAN	*g*	c
0010	Stupicky Hanacky	CSK	*a8*	b
0007	Stupicky Plnozrnny	CSK	*a8*	b
1046	Sudan	USA	none	b
1165	Sulu	AUS	*k1*	a
0383	Tamina	DDR	*a13*	a
1339	Tellus	SWE	*g*	a
1339	Tellus	SWE	*a12*	e
0548	Thaya Loosdorfers	AUT	*a8*	b
2376	Torcal	ESP	*g*, *u*	a
0234	Trebi	USA	*a8*	b
1097	Triple Awn Lemma	USA	*Ch*	e
0011	Triumf	CSK	*a8*	b
0011	Triumf	CSK	none	b
1019	Trumpf	DDR	*a7*, *k1*, *La*	d
1019	Trumpf	DDR	*a13*, *g*	e
1019	Trumpf	DDR	*a9*	e
0572	Tschermaks	AUT	*Ch*, *He2*	b
0572	Tschermaks	AUT	none	b
0572	Tschermaks	AUT	*g*, *He2*	d
1969	Turk	TUR	none	b
1969	Turk	TUR	*IM9*, *Lo*	e
0262	Umanskij	SUN	none	b
0564	Union Firlbecks	DEU	*g*, *He2*	a
0019	Valticky	CSK	*a8*, *He2*	b
0880	Varde	NOR	none	b
1651	Vega Abed	DNK	*a13*	e
0264	Viner	SUN	*a8*	b
0264	Viner	SUN	none	b
0264	Viner	SUN	*g*	e
2364	Viva	AUT	*a9*, *u*	a
1251	Voldagsen ST. 824/44	DEU	*u*	d
1251	Voldagsen ST. 824/44	DEU	*a9*	e
2328	Vybor	SUN	*a8*	d
2328	Vybor	SUN	none	d
0521	Weihenstephaner Mehltauresistante	DEU	*g*	a
0562	Wisa Breuns	DEU	*g*, *He2*	a
0842	Wong	CHN	*g*	d
0849	Woodrow	USA	*a8*	b
0707	Ymer	SWE	*a8*	a
0037	Zidlochovicky Gloria	CSK	*a8*	b

^a^Identification number of the Czech gene bank of spring barley.

^b^Country of origin: ARG—Argentina, AUS—Australia, AUT—Austria, BEL—Belgium, BOL—Bolivia, CAN—Canada, CSK—Czechoslovakia, CZE—Czech Republic, DDR—East Germany, DEU—Germany, DNK—Denmark, DZA—Algeria, EGY—Egypt, ESP—Spain, ETH—Ethiopia, FIN—Finland, FRA—France, GBR—Great Britain, GRC—Greece, HUN—Hungary, CHN—China, IND—India, IRL—Ireland, JPN—Japan, LVA—Latvia, MNG—Mongolia, NLD—Netherlands, NOR—Norway, POL—Poland, SUN—Soviet Union, SWE—Sweden, TUR—Turkey, USA—United States of America, YUG—Yugoslavia.

^c^Category: a—genotypes whose identified resistance was consistent with published data; b - genotypes for which the observed resistance is probably consistent with previous data, and those for which there were no data to indicate an erroneous designation; c–insufficient data to validate genotype identity; d—genotypes for which the data indicate a discrepancy in genotype authenticity; e—genotypes whose recorded resistance is inconsistent with published data.

In total there were 63 RTAs (excluding 27 RTAs that had unknown resistances) and 13 isolates were sufficient to separate them (S2 Table). Twenty-two known *Ml* resistance genes (*a1*, *a3*, *a6*, *a7*, *a8*, *a9*, *a12*, *a13*, *Ab*, *at*, *g*, *h*, *He2*, *Ch*, *IM9*, *k1*, *La*, *Lo*, *mlo*, *p1*, *ra* and *Ru2*), occurring either separately or in combinations were identified. Among the most frequent resistance genes found in 327 genotypes were *Mla8* (in 99 genotypes) and *Mlg* (in 75 genotypes); 43 genotypes contained no resistance genes (= none). We also observed a higher frequencies of *Ml* genes *La* (32), *He2* (29), *Ch* (24), *a7* (22), *k1* (21) and *a13* (20). The total frequency of the known genes determined in all 327 genotypes was 406. In addition, in 27 of these genotypes we noted an unknown resistance combined with at least one (18 cases) and, in three cases, two known resistance genes. In some genotypes, we detected “additional” *Ml* genes closely linked to alleles of the *Mla* locus (*aAl2*, *a14*, *aEm2*, etc.). Such genes are not shown and discussed further because in most of the remaining genotypes that were expected to contain “additional” genes, this could not be conclusively established.

All 327 genotypes were divided into five categories, of which the first category (a) includes the genotypes whose identified resistance was consistent with published data (97 genotypes). The second category (b) of 109 genotypes were those whose determined resistance is consistent with the resistance of the given variety (e.g. ‘none’ resistance gene or *Mla8* in the case of the older varieties), and those for which there were no data challenging their identity. The third category (c) is represented by 30 genotypes for which there are no previous published data. The fourth category (d) includes 28 genotypes where there are doubts about their authenticity, and the fifth category (e) comprises 63 genotypes whose resistance is inconsistent with published data.

Among 223 accessions of the collection, we found 151 homogeneous accessions, but the resistance of nine of them was inconsistent with published data, and 12 of those remaining have doubtful authenticity. In 72 heterogeneous accessions represented by 176 accession × powdery mildew resistance genotypes, 54 genotypes had a resistance that is inconsistent with published data. These have clearly resulted from mechanical admixtures. Regarding the other 16 heterogeneous genotypes there are doubts as to their authenticity.

## Discussion

The first European commercial variety of spring barley intentionally bred for the incorporation of a mildew resistance (*Mlg*), was the German variety **Union** registered in 1955 [18]. Union was followed by varieties possessing other specific resistance genes of which there are now several dozens. These are present either singly or in combinations [19,26] and have influenced the composition and increased complexity of the Central European population of the pathogen [27,28]. In 1979 the first commercial variety (**Atem**) with the *mlo* non-specific resistance gene was registered [29] and this resistance has become dominant in spring barley varieties [21,26]. Thus, barley resistance to powdery mildew conditioned by many major genes is highly diverse with a progressive utilisation of individual genes in commercial varieties that has been extensively reported.

### Discrepancies among homogeneous accessions

In **Triple Awn Lemma**, *MlCh* was found, which is completely ineffective except against one isolate that we used, while Nover and Lehmann [30] recorded high resistance in this variety conditioned by a combination of the *Mla9* and *Mlk1* genes [14]. In **Archer, Maskin** and **Plumage Archer**, we identified *Mla8*, but in the catalogue [19] there is no mention of a resistance gene (= none). The results included in the catalogue are based on the study that focused particularly on the detection of *Mla8* [31]. In **Vega Abed**, *Mla13* was uncovered while the catalogue states *MlLa*, and in **Rupee**, which is a known source of the *Ml* genes, *a13* (= *aRu1*), *Ru2*, *aRu3*, *aRu4*, a different unknown resistance was detected. In the Australian variety **Malebo**, we established the presence of *Mla8* and *Mlk1*, while Dreiseitl and Platz [23] found only *Mla8*. It is possible that Malebo was composed of two lines, one of which was described in the previous research and the other was the one we investigated. **Otra** contained *Mla7* and *MlLa*, while this variety was reported as being susceptible in Latvia [32], and confirmed by Hovmøller et al. [33]. **Odesskij 9** is a selection from an unknown variety which was acquired for the gene bank in 1958. The fact that we found both *Mlg* and *MlLa* in this variety poses questions about its authenticity as the first known variety with *MlLa* (Vada) was registered in 1963.

### Discrepancies among heterogeneous accessions

We identified 37 heterogeneous accessions with incorrect genotypes. In this report we will focus on six accessions in which none of the genotypes consistently corresponded with previous data. Progenies of **Abyssinian 1102** contained three genotypes, but none of them possesses *mlo*, which is present in the genuine Abyssinian 1102 [29]. Furthermore, *mlo* is often naturally present only in Ethiopian barleys. In accessions marked as **Asse**, we found two genotypes, but neither carried *Mlg* specified in the catalogue. Similarly, two genotypes were uncovered in **Emir**, neither of which was *Mla12*, although Emir is known as a source of the latter, and the accepted code of this resistance (Em) was derived from this variety. Moreover, although in **Gerda** *Mla6* is listed together with *Mlg* in the catalogue we did not find evidence to confirm this. **KM 1192** is the original source of the resistance used for the first time in Kredit after which the resistance is named *MlKr* [20]. However, in the KM 1192 accession we recorded two different lines (*Mla8* and *Mla8*, *MlLa*). In **Opal** (Czech), there were two genotypes (*Mla7*, *MlLa* and *Mla8*), while the original one contained *Mla6* and *MlLa* [20]. *Mla8* is present in a number of varieties, for example in Danish Opal [19].

### Identical designation of different varieties

**Sarah,** which originated from France and was described as an alternative rather than spring type, was lodged in the gene bank in 1974. We obtained no evidence of a resistance gene, which could be supported by the fact that Sarah was selected from Champagner. In England, *Mla12* was reported in winter Sarah [34], and in Germany an unknown resistance was observed possibly in another winter form of Sarah [35].

In **Commander**, deposited in the gene bank in 1958, *Mla8* and another unknown resistance was revealed. In a set of Australian barleys a variety with the same name was studied [23]. However, it was registered much later (2004) and its two lines carried *Mlg*, *MlGa* and *Mlg*, *MlLa*.

**Wong** (China) is a known source of the resistance gene that is named after it–*MlWo* [36]. On the other hand, there are spring and winter varieties also known as Wong and it is not clear which of them is the true source of this gene. Schwarzbach and Fischbeck [18] identified *MlWo* in two winter varieties, whereas in our tests Wong carried *Mlg*.

No specific resistance gene was found in either **Manchuria** or **Oderbrucker**, which is a selection from Manchuria. In Poland Manchuria was used in the pathogen survey as a susceptible variety [33]. On the other hand, Wiberg [14] states that Manchuria (C.I. 2610) has genes that are identical with those in Algerian (*Mla1*, *Mlat*). Therefore, Manchuria that was the subject of our research and in Poland, as well as the Manchuria from which Oderbrucker was selected, differs from the Manchuria studied by Wiberg [14].

In **Esperance No. 227/1960**, we detected *Mla8*, while Brückner [13] and Schwarzbach and Fischbeck [18] mention that Esperance has a typical and phenotypically very different resistance gene. It seems that Esperance and Esperance No. 227/1960 are different varieties.

### Anomalies

#### Adonia

According to the catalogue [19], Adonia as well as its parents are winter types. We found four genotypes with the following *Ml* resistance genes: *a6*, *h*, *ra*; *p1*; *p1*, *at* and *a12*, *u*. The pedigree of Adonia is Espe × Stamm729 × Vogelsanger Gold × Inka. Schwarzbach and Fischbeck [18] studied Adonia and reported a combination of *Mla6* and *Mlh*. The catalogue mentions the resistance of their three parents (Espe-*Mlra*, Inka-*Mlh* and Vogelsanger Gold-*Mla6*, *Mlh*, *Mlra*). The combination of *Ml* genes specified for Adonia thus corresponds to the genes carried by two of the parents and is identical to that (*Mla6*, *Mlh*, and *Mlra*) in one of the three characterised genotypes [37] and in one of the four genotypes studied here. However, all these genes occur more frequently in winter rather than spring varieties [38].

*Mlp1*, which was present in two Adonia genotypes and two of the three previously described genotypes [37], is one of the oldest known resistance genes [12], although its presence in commercial varieties is rare. This gene was also detected in one of the three genotypes of Seljanin (*Mlp1*, *Mla6*) whose parent is Adonia (Adonia × Perf × Muronec). We can confirm, therefore, the presence of *Mlp1* in both Adonia and its daughter Seljanin. Nevertheless, the question of why the detection or specification of the Adonia line carrying *Mlp1* was not mentioned by Schwarzbach and Fischbeck [18] remains open.

#### Hanna

We recorded the presence of *Mlg* in Hanna and Binder Abed (a selection from Hanna bred in 1913). Nover and Lehmann [30] also state that Hanna (C.I. 906) contains *Mlg*. C.I. 906 is a selection from C.I. 34 (Hanna pedigree) which was collected in Austria in 1900 (at that time the Czech Republic was a part of the Austrian empire). Also in Selekcni Hanacky VIII, which is again a selection of the original regional Hana variety (Hanna), three genotypes were found, two of which carry *Mlg*. However, the catalogue states that *Mla8* is in both these varieties.

The name Hanna (Hana) is derived from the name of a fertile region of the Czech Republic (Haná) and traditionally an area where high quality malting barleys have been grown. Therefore, the name has been assigned to several varieties of different crop species including barley. The Hanna carrying the resistance gene that was named after this variety, *Mlh* [14], and Heils Hanna carrying *Mla8* [36], after which the code of this resistance (HH) was named, belong to this group.

In 1973 another derivative Hana, in which no resistance gene was recorded [20], but which could carry *Mla8*, was registered in the Czech Republic. This Hana was screened by us and we uncovered two genotypes, namely one with *Mla8* (which is regarded as genuine) and the other with *Mlg*, which had not been found in this variety before [20].

In Hanna, we confirmed the presence of *Mlg* found in this variety by Nover and Lehmann [30]. We also detected *Mlg* in selections from Hanna (Hana), namely Binder Abed and Selekcni Hanacky VIII. It seems highly likely that the Hanna we tested did possess *Mlg* and could be one of the original sources of this gene revealed here.

#### Nadja and Trumpf

For Nadja, Brown and Jørgensen [19] note the presence of *Mla7* and Trumpf is named Triumph with the genes *Mla7*, *MlAb*, and *MlTr3*. We uncovered two genotypes for Nadja together with four genotypes in the Trumpf accession. In each of these varieties there was one genotype carrying *Mla7* and in both there was an identical combination of *Mla7*, *Mlk1*, and *MlLa*, which differs from the catalogue data.

## Conclusions

The goal of our study of heterogeneous accessions was to identify the resistance(s) contained in these accessions. By examining five individually harvested plants of each accession we reliably established all resistances, but we could not find genotypes that occurred less frequently. This explains why we came across identical resistances in each of the 61 sets of plant progenies of the 133 heterogeneous accessions.

Dreiseitl [39] studied heterogeneous wild barleys (*H*. *vulgare* subsp. *spontaneum*) maintained in the ICARDA gene bank. For each of the 128 accessions five plant progenies were tested. Forty-four accessions were composed of two genotypes, 25 accessions of three genotypes, 10 accessions of four and two accessions comprised five genotypes. A total of 260 genotypes were found, equalling 2.03 genotype per accession. We tested 133 accessions in the same manner and detected 237 genotypes, i.e. 1.78 genotype per accession on average.

Wild barley is well-known for its high resistance diversity [39–43] and its diversity in the gene bank might have arisen from collecting bulked heterogeneous samples along with outcrossing in the field because of its open flowering nature [44]. It is surprising, therefore, that the value of the average number of genotypes in one accession of the core collection (1.78) was similar to the value in the collection of wild barley (2.03).

The most frequent gene found in 99 genotypes was *Mla8*, which is detectable only with pathotypes appearing in Japan [45]. The actual frequency of *Mla8* must be even higher since only Race 1, which is avirulent to many specific resistance genes including *Mlg*, was available for its detection. *Mla8* is often accompanied by *MlHe2*—we revealed this combination in 15 genotypes. However, in nine genotypes with *MlHe2* we also found *Mlg*, which masks *Mla8*. Hence, in these nine genotypes *Mlg* and *MlHe2* could be accompanied by *Mla8*. The latter gene could also be present in the absence of *MlHe2* in some genotypes containing *Mlg*.

Jørgensen and Jensen [31] studied the presence of *Mla8* in 63 European varieties of spring barley bred in the first half of the 20th century and identified *Mla8* in 40 of them. In addition, *Mla8* occurs frequently in Australian [23] and Chinese varieties [46] and elsewhere. As well as this gene and in the absence of any specific resistance gene (none), older varieties of spring barley may naturally have carried *MlHe2* and *MlCh*, and South Asian barleys [46] possess *MlRu2* too (formerly designated as *MlBw*). The older varieties were often bred by bulk selection from landraces or after cross-breeding and no subsequent selection for undetected resistances. This explains why the existence of two or more genotypes (lines) may not be mechanical admixtures but may be an inherent feature of these varieties. A good example is the domestic landrace Dobrovicky Starocesky, in which there were four genotypes (none, *MlCh*, *Mla8* and *Mla8*, *MlHe2*) and all of them could be considered as the original progenies.

Plant progenies used in this research will serve as the basis for multiplying genotypically pure varieties. In the future we will replace accessions that are not genuine, and whose authenticity is in doubt, with well-characterised accessions from other gene banks. We will then test them using similar methods to verify their identity. Accessions with unknown resistances will be subject to further studies.

Our investigation of the core collection has confirmed earlier findings that accessions in gene banks are often contaminated or even confused with other genotypes [4]. In addition, we have demonstrated that identifying barley resistance genes to powdery mildew is an effective although not totally reliable tool that can reveal such errors. To expand our abilities, there are several pathogens of cereals, particularly rusts and mildews, against which many resistance genes in host crops have also been utilized [47–51]. Knowledge and identification of these genes can lead to the purification of accessions in gene banks. Seed purity and accession authenticity can subsequently be checked by more advanced and less laborious methods.

## Supporting information

S1 Table Origin of 64 *Blumeria graminis* f. sp. *hordei* isolates used for response tests of 223 varieties in the Czech spring barley core collection. (DOC)

S2 Table Sixty-three response type arrays produced by 13 selected *Blumeria graminis* f. sp. *hordei* isolates on 223 varieties of the Czech spring barley core collection. (DOC)
