# Supplementary material for: Identification of barley powdery mildew resistances in gene bank accessions and the use of gene diversity for verifying seed purity and authenticity
Source: PLoS One. 2018 Dec 7;13(12):e0208719. doi: 10.1371/journal.pone.0208719 (PMC6285996; doi:10.1371/journal.pone.0208719)
Supplement: S1 Table — (DOC) [file pone.0208719.s001.doc]

Supplementary material

**Identification of barley powdery mildew resistances in gene bank accessions and the use of gene diversity for verifying seed purity and authenticity**

Antonín Dreiseitl^1*^ and Marta Zavřelová^2¶^

*** Correspondence:** Antonín Dreiseitl, [dreiseitl@vukrom.cz](mailto:dreiseitl@vukrom.cz)

**S1 Table.** Origin of 64 *Blumeria graminis* f. sp. *hordei* isolates used for response tests of 223 varieties in the Czech spring barley core collection

| A-GH/05^a^ | U-54/05 | CR-63/97 | CR-I162/09 | CR-E6/11 | CR-I6/14 |
| --- | --- | --- | --- | --- | --- |
| A-11/655 | US-A/16 | CR-A39/99 | CR-I167/09 | CR-I20/11 | CR-M3/14 |
| CH-3-33/03 | US-B/16 | CR-C73/99 | CR-K200/09 | CR-A7/12 | CR-Z6/14 |
| I-35/79 | DK-86/b93 | CR-C512/01 | CR-L209/09 | CR-I16/12 | CR-A1/15 |
| I-69/79 | G-120/b99 | CR-C132/02 | CR-M236/09 | CR-X30/12 | CR-A2/15 |
| I-148/79 | F-18/b95 | CR-J20/04 | CR-Y2/10 | CR-C1/13 | CR-K3/15 |
| I-462/79 | SW-18/75 | CR-D48/07 | CR-E2-2/10 | CR-L1/13 | CR-KM6/15 |
| I-16/97 | SW-30/b76 | CR-F72/07 | CR-X4/10 | CR-L11/13 | CR-M4/15 |
| J-Race I/53 | SW-26/76 | CR-C109/08 | CR-F8/10 | CR-Y4/13 | CR-O11/16 |
| JAR-65/04 | CR-4776/b92 | CR-A23/09 | CR-C10/10 | CR-B2-1/14 |  |
| JAR-4-20/07 | CR-21/97 | CR-B45/09 | CR-K2/11 | CR-C2/14 |  |

^a^A = Australia, CH = China, CR = Czech Republic, DK = Denmark, G = Germany, F = France, I = Israel, J = Japan, JAR = Republic of South Africa, SW = Sweden, U = Uruguay, US = United States of America; numbers after a slash (/) indicate the year of isolate collection and b = before that year.
