# Supplementary material for: Identification of barley powdery mildew resistances in gene bank accessions and the use of gene diversity for verifying seed purity and authenticity
Source: PLoS One. 2018 Dec 7;13(12):e0208719. doi: 10.1371/journal.pone.0208719 (PMC6285996; doi:10.1371/journal.pone.0208719)
Supplement: S2 Table — (DOC) [file pone.0208719.s002.doc]

Supplementary material

**Identification of barley powdery mildew resistances in gene bank accessions and the use of gene diversity for verifying seed purity and authenticity**

Antonín Dreiseitl^1*^ and Marta Zavřelová^2¶^

*** Correspondence:** Antonín Dreiseitl, [dreiseitl@vukrom.cz](mailto:dreiseitl@vukrom.cz)

**S2 Table.** Sixty-three response type arrays produced by 13 selected *Blumeria graminis* f. sp. *hordei* isolates on 223 varieties of the Czech spring barley core collection

| *Ml* resistance |  |  |  |  |  |  | Isolate |  |  |  |  |  |  |
| --- | --- | --- | --- | --- | --- | --- | --- | --- | --- | --- | --- | --- | --- |
| gene(s) | DK-86 | I-462 | Race I | SW-26 | SW-30 | U-54 | 4776 | A-7 | C-132 | D-48 | I-16 | L-1 | X-30 |
| none | 4^a^ | 4 | 4 | 4 | 4 | 4^1^ | 4 | 4 | 4 | 4 | 4 | 4 | 4 |
| *a1*, *aAl2* | 0 | 4 | 1-2 | 0 | 0 | 0 | 0 | 4 | 4 | 0 | 4 | 0 | 4 |
| *a1*, *aAl2*, *Ab* | 0 | 2 | 1-2 | 0 | 0 | 0 | 0 | 4 | 2 | 0 | 4 | 0 | 4 |
| *a1*, *at* | 0 | 4 | 1-2 | 0 | 0 | 0 | 0 | 2 | 4 | 0 | 2 | 0 | 2 |
| *a1*, *aAl2*, *g* | 0 | 4 | 0 | 0 | 0 | 0 | 0 | 4 | 4 | 0 | 4 | 0 | 4 |
| *a1*, *g*, *k1* | 0 | 4 | 0 | 0 | 0 | 0 | 0 | 4 | 2 | 0 | 2 | 0 | 2 |
| *a1*, *aAl2*, *La* | 0 | 4 | 1-2 | 0 | 0 | 0 | 0 | 2-3 | 4 | 0 | 4 | 0 | 2-3 |
| *a3* | 4 | 4 | 1 | 1 | 1 | 1 | 1 | 1 | 4 | 1 | 1 | 1 | 4 |
| *a3*, *g* | 0 | 4 | 0 | 1 | 0 | 1 | 0 | 1 | 4 | 1 | 1 | 1 | 4 |
| *a3*, *g*, *La* | 0 | 4 | 0 | 1 | 0 | 1 | 0 | 1 | 4 | 1 | 1 | 1 | 2-3 |
| *a6*, *a14* | 4 | 4 | 0 | 0 | 2-3 | 0 | 4 | 4 | 4 | 4 | 4 | 4 | 4 |
| *a6*, *g* | 0 | 4 | 0 | 0 | 0 | 0 | 0 | 4 | 4 | 4 | 4 | 4 | 4 |
| *a6*, *h*, *ra* | 4 | 4 | 0 | 0 | 0-1 | 0 | 4 | 4 | 1-2 | 4 | 4 | 4 | 4 |
| *a6*, *La* | 4 | 4 | 0 | 0 | 2-3 | 0 | 4 | 2-3 | 4 | 2-3 | 4 | 2-3 | 2-3 |
| *a7*, *aNo3* | 1-2 | 1-2 | 0 | 1-2 | 1-2 | 1-2 | 4 | 1-2 | 4 | 4 | 4 | 4 | 4 |
| *a7*, *aNo3*, *aNo4*, *k1* | 1-2 | 1-2 | 0 | 1-2 | 1-2 | 1-2 | 4 | 1-2 | 1-2 | 2 | 2 | 2 | 1-2 |
| *a7*, *aNo3*, *g* | 0 | 1-2 | 0 | 1-2 | 0 | 1-2 | 0 | 1-2 | 4 | 4 | 4 | 4 | 4 |
| *a7*, *aNo3*, *g*, *k1* | 0 | 1-2 | 0 | 1-2 | 0 | 1-2 | 0 | 1-2 | 2 | 2 | 2 | 4 | 2 |
| *a7*, *aNo3*, *g*, *La* | 0 | 1-2 | 0 | 1-2 | 0 | 1-2 | 0 | 1-2 | 4 | 2-3 | 4 | 2-3 | 2-3 |
| *a7*, *aNo3*, *h*, *La* | 1-2 | 1-2 | 0 | 1-2 | 1-2 | 1-2 | 4 | 1-2 | 1-2 | 4 | 4 | 4 | 4 |
| *a7*, *aNo3*, *k1* | 1-2 | 1-2 | 0 | 1-2 | 1-2 | 1-2 | 4 | 1-2 | 2 | 2 | 2 | 4 | 2 |
| *a7*, *aNo3*, *k1*, *La* | 1-2 | 1-2 | 0 | 1-2 | 1-2 | 1-2 | 4 | 1-2 | 2 | 2 | 2 | 2-3 | 2 |
| *a7*, *aNo3*, *La* | 1-2 | 1-2 | 0 | 1-2 | 1-2 | 1-2 | 4 | 1-2 | 4 | 2-3 | 4 | 2-3 | 2-3 |
| *a8* | 4 | 4 | 0 | 4 | 4 | 4 | 4 | 4 | 4 | 4 | 4 | 4 | 4 |
| *a8*, *He2* | 4 | 4 | 0 | 4 | 4 | 2-3 | 4 | 4 | 4 | 4 | 4 | 4 | 4 |
| *a8*, *k1* | 2 | 4 | 0 | 4 | 2 | 4 | 4 | 4 | 2 | 2 | 2 | 4 | 2 |
| *a8*, *k1*, *La* | 2 | 4 | 0 | 2-3 | 2 | 4 | 4 | 2-3 | 2 | 2 | 2 | 2-3 | 2 |
| *a8*, *La* | 4 | 4 | 0 | 2-3 | 2-3 | 4 | 4 | 2-3 | 4 | 2-3 | 4 | 2-3 | 2-3 |
| *a9* | 0 | 0 | 0 | 4 | 0 | 0 | 4 | 0 | 0 | 4 | 0 | 0 | 0 |
| *a9*, *g* | 0 | 0 | 0 | 4 | 0 | 0 | 0 | 0 | 0 | 4 | 0 | 0 | 0 |
| *a9*, *k1* | 0 | 0 | 0 | 4 | 0 | 0 | 4 | 0 | 0 | 2 | 0 | 0 | 0 |
| *a9*, *k1*, *La* | 0 | 0 | 0 | 2-3 | 0 | 0 | 4 | 0 | 0 | 2 | 0 | 0 | 0 |
| *a12* | 1 | 4 | 1 | 1 | 4 | 1 | 4 | 4 | 4 | 4 | 4 | 4 | 4 |
| *a12*, *g* | 0 | 4 | 0 | 1 | 0 | 1 | 0 | 4 | 4 | 4 | 4 | 4 | 4 |
| *a12*, *g*, *k1* | 0 | 4 | 0 | 1 | 0 | 1 | 0 | 4 | 2 | 2 | 2 | 4 | 2 |
| *a12*, *g*, *La* | 0 | 4 | 0 | 1 | 0 | 1 | 0 | 2-3 | 4 | 2-3 | 4 | 2-3 | 2-3 |
| *a12*, *La* | 0 | 4 | 1 | 1 | 2-3 | 1 | 0 | 2-3 | 4 | 2-3 | 4 | 2-3 | 2-3 |
| *a13* | 0 | 0 | 0 | 4 | 0 | 0 | 4 | 4 | 4 | 4 | 0 | 4 | 4 |
| *a13*, *g* | 0 | 0 | 0 | - | 0 | 0 | 0 | - | 4 | 4 | 0 | 4 | 4 |
| *a13*, *La* | 0 | 0 | 0 | - | 0 | 0 | 4 | - | 4 | 2-3 | 0 | 2-3 | 2-3 |
| *a13*, *g*, L*a* | 0 | 0 | 0 | - | 0 | 0 | 0 | - | 4 | 2-3 | 0 | 2-3 | 2-3 |
| *at* | 2 | 4 | 2 | 2 | 2 | 2 | 4 | 2 | 4 | 2 | 2 | 2 | 2 |
| *g* | 0 | 4 | 0 | 4 | 0 | 4 | 0 | 4 | 4 | 4 | 4 | 4 | 4 |
| *g*, *at* | 0 | 4 | 0 | 2 | 0 | 2 | 0 | 2 | 4 | 2 | 2 | 2 | 2 |
| *g*, *h* | 0 | 4 | 0 | 4 | 0 | 1-2 | 0 | 4 | 1-2 | 4 | 4 | 4 | 4 |
| *g*, *He2* | 0 | 4 | 0 | 4 | 0 | 2-3 | 0 | 4 | 4 | 4 | 4 | 4 | 4 |
| *g*, *k1* | 0 | 4 | 0 | 4 | 0 | 4 | 0 | 4 | 2 | 2 | 4 | 4 | 2 |
| *g*, *La* | 0 | 4 | 0 | 2-3 | 0 | 4 | 0 | 2-3 | 4 | 2-3 | 4 | 2-3 | 2-3 |
| *g*, *Lo* | 0 | 0 | 0 | 4 | 0 | 4 | 0 | 4 | 4 | 4 | 4 | 4 | 4 |
| *g*, *Lo*, *He2* | 0 | 0 | 0 | 4 | 0 | 2-3 | 0 | 4 | 4 | 4 | 4 | 4 | 4 |
| *h* | 4 | 4 | 4 | 4 | 4 | 1-2 | 4 | 4 | 1-2 | 4 | 4 | 4 | 4 |
| *Ch* | 4 | 4 | 2 | 4 | 4 | 4 | 4 | 4 | 4 | 4 | 4 | 4 | 4 |
| *Ch*, *He2* | 4 | 4 | 2 | 4 | 4 | 2-3 | 4 | 4 | 4 | 4 | 4 | 4 | 4 |
| *Ch*, *ra* | 4 | 4 | 2 | 4 | 0-1 | 4 | 4 | 4 | 4 | 4 | 4 | 4 | 4 |
| *IM9*, *Lo* | 1 | 0 | 0 | 1 | 1 | 1 | 1 | 4 | 1 | 4 | 1 | 4 | 1 |
| *k1* | 2 | 4 | 2 | 4 | 2 | 4 | 4 | 4 | 2 | 2 | 2 | 4 | 2 |
| *Lo*, *ra* | 4 | 0 | 0 | 4 | 0-1 | 4 | 4 | 4 | 4 | 4 | 4 | 4 | 4 |
| *mlo* | 0(3) | 0(3) | 0(3) | 0(3) | 0(3) | 0(3) | 0(3) | 0(3) | 0(3) | 0(3) | 0(3) | 0(3) | 0(3) |
| *p1* | 2 | 4 | 2 | 2 | 2 | 2 | 2 | 2 | 2 | 2 | 2 | 4 | 4 |
| *p1*, *at* | 2 | 4 | 2 | 2 | 2 | 2 | 2 | 2 | 2 | 2 | 2 | 2 | 2 |
| *p1*, *g* | 0 | 4 | 0 | 2 | 0 | 2 | 0 | 2 | 2 | 2 | 2 | 4 | 4 |
| *p1*, *Lo*, *ra* | 2 | 0 | 0 | 2 | 0-1 | 2 | 2 | 2 | 2 | 2 | 2 | 4 | 4 |
| *Ru2* | 2-3 | 4 | 4 | 4 | 4 | 2-3 | 4 | 4 | 4 | 2-3 | 2-3 | 2-3 | 4 |

^a^Response types 0 - 4, where 0 = resistant and 4 = susceptible.
